# Misconduct in Research and Publication: a Dilemma That Is Taking Place

**Published:** 2017-07

**Authors:** Mohammad Hossein Asghari, Milad Moloudizargari, Mohammad Abdollahi

**Affiliations:** 1 Department of Pharmacology, Faculty of Medicine, Babol University of Medical Sciences, Babol, Iran; 2 Department of Toxicology and Pharmacology, Faculty of Pharmacy, Tehran University of Medical Sciences, Tehran, Iran; 3 Student Research Committee, Department of Immunology, School of Medicine, Shahid Beheshti University of Medical Sciences, Tehran, Iran; 4 Toxicology and Diseases Group, Pharmaceutical Sciences Research Group, Tehran University of Medical Sciences, Tehran, Iran

Having considered current reports concerning plagiarisms taking place in the global science community, the authors decided to address the principal reasons, which lead to these illegalities. In recent years, misconduct in research, such as plagiarism, fabrication, falsification, guest author, ghost author, self-citation, etc. have been increasing significantly in scientific papers, proving a lack of commitment to publication ethics among some authors.

## Consequences of plagiarism

Despite the scarce number of such papers, they may bring along consequent detrimental effects with a high possibility of irreparable results. Each paper, which is a sole compilation of plagiarized data, puts the science community’s prestige in great jeopardy, and at the same time all the investments, finances, and time, put behind that, are down the drain. To make things worse, such papers may serve as the basis of more researches with a horizon of alarmingly irretrievable losses. What if instead of investing on such a wrong way, which is basically for one’s self-interest rather than the promotion of the scientific community, all of these investments were poured into research and endeavor contributing to solutions for society’s problems. Loss of trust and confidence in science community is the worst repercussion of plagiarism, which makes researchers have grave doubts in all scientific findings and always see a question mark regarding almost any paper they may encounter. It seems as if, all deterring rules, regulations and fines, aimed at creating barriers on the way of perpetrators, have not obstructed their progress, and more and more of the above papers are increasing[1,2].

## Current concerns from authors’ point of view

Ph.D. candidates and masters level students, who by force of educational rules, are required to publish articles during their educational courses and long for non-scientific ways to lead the rest of their lives, are among other victims of some erroneous policies forcing them to write papers as a demanding job. This, indeed, pushes them towards disregard for legal frameworks of publication ethics[3].

Impact factor (IF) is a scientifically almost agreed measure for determining the rank of journals, although some new and better ones are being introduced. This index has limitations and does not necessarily reflect the credit of a journal. Although it might seem a good option; however, principle reconsiderations are required so that all aspects of a scientifically proven journal are covered. For instance, a sole article that might receive a high number of citations may easily affect a journal’s IF but it does not mean that other articles in that journal have the same quality as the mentioned paper[2]. Sometimes, some articles with an unbelievable number of authors are observed that are published in even good journals. The first question comes to mind is that how is exactly possible that one article may have more than 100 or even many more and whether it is consistent with ICMJE (http://www.icmje.org/recommendations/browse/ roles-and-responsibilities/defining-the-role-of-authors-and-contributors.html) criteria for authorship. At the moment, we are not in the opinion that all huge multiauthor papers are inconsistent with ICMJE criteria, but this can be a subject of discussion. The other concern is that such articles may simply disrupt the fundamental principles of citation measures and related parameters.

One of the biggest issues that researchers are dealing with is their insistence on all papers producing positive outcomes. Most of them wrongly think in a way that setting up and advocating a research proposal equal positive results. Although a publication with negative results might seem ineffectual at the first glance; however, it may shed light on the topics and the originality of previous publications reporting positive results[4]. This forces the researchers to expect only positive result, which could be the cause of bias in the way of conducting the research, *per se*. Such a misunderstanding will undeniably impose a negative impact on researchers’ findings and interpretations. The worse is the eagerness of a great variety of journals to only publish positive results. On such a basis, the question that comes to mind is that if all researches were supposed to create positive results, then why are we yet carrying out research to this large extent?

Even though hypothesis is the essence of every paper, it does not mean it should always come to fully satisfying results. Research might be done for years, revealing all the previously performed studies to be wrong. Just because a well-known researcher’s work has to come to a desired result, shouldn’t make you be dubious about your results; as not only is there a possibility that the researcher has unwittingly made a mistake, but many other factors may have also played roles in that result. On the other hand, the science advances and laboratory methods are proving more meticulous, leading to extensive changes in previous knowledge[5].

Supervisors, who would not disgruntle hearing “this substance didn’t work” after a course of two-year research, are in the minority. The majority of them, in response to such a statement, would compare it with the results of the research already conducted by experts and may hold their fellows accountable for the failure of their research and this might serve as the beginning of plagiarisms and detours in question.

Another concern is whether or not labeling research with terms of immorality would help to solve problems or may it harm the prestige of scientific community? The point is that although making these problems public may be in some ways harmful; however, revealing different aspects of misconduct can be helpful in eliminating its worst consequences. Moreover, discussing the issues of research misconduct in more private sessions may protect the outlook of the scientific community of medical and health care systems in the public.

True researchers, who are not few in number, never turn to wrong ways to achieve privileges and deserve a big mention of name here. There could be hope that the scientific community never sees these immoralities. Apparently, the science world policies are in a great need of reforming criteria regarding publication ethics. Research is not comparable with soccer, in which fans’ morale is either boosted or destroyed when scoring or conceding a goal. However, conversely, conducting a medical research resembles walking on a narrow bridge built on a deep canyon, on which a misplacement of one step may lead to countless death, since researches are cornerstone of medical treatment and remedies.
